# Supplementary material for: Transcriptomic Insights into Mechanisms of Early Seed Maturation in the Garden Pea (Pisum sativum L.)
Source: Cells. 2020 Mar 23;9(3):779. doi: 10.3390/cells9030779 (PMC7140803; doi:10.3390/cells9030779)

Zhongwan−6,10(4)

Global Summary

%DE = 0.27

genes with fdr < 0.2 = 169 (77 + / 92 −)

genes with fdr < 0.1 = 144 (63 + / 81 −)

genes with fdr < 0.05 = 137 (60 + / 77 −)

genes with fdr < 0.01 = 123 (53 + / 70 −)

genes in genesets = 0

<FC> = 0

<t−score> = 0

<p−value> = 0

<fdr> = 0.73

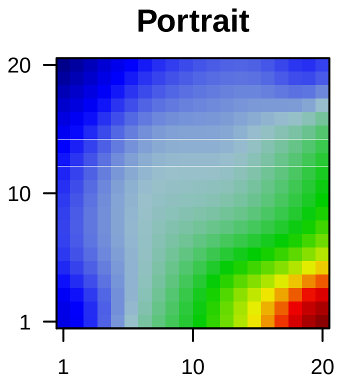

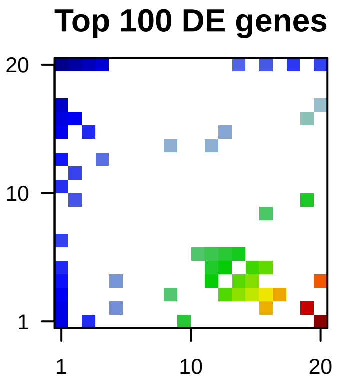

Global Genelist

Rank	ID	log(FC)	p−value	fdr	Metagene	Description
1	TRINITY_DN2	0.85	2e−16	1e−15	14 x 3	
2	TRINITY_DN4	0.25	2e−16	1e−15	12 x 4	
3	TRINITY_DN1	−0.38	2e−16	1e−15	1 x 2	
4	TRINITY_DN1	5.71	2e−16	1e−15	20 x 1	
5	TRINITY_DN1	−3.79	2e−16	1e−15	20 x 20	
6	TRINITY_DN5	−0.4	2e−16	1e−15	2 x 20	
7	TRINITY_DN3	−0.21	2e−16	1e−15	1 x 15	
8	TRINITY_DN5	−0.31	2e−16	1e−15	1 x 11	
9	TRINITY_DN2	−0.18	2e−16	1e−15	20 x 1	
10	TRINITY_DN2	−1.19	2e−16	1e−15	20 x 1	
11	TRINITY_DN3	−0.32	2e−16	1e−15	1 x 1	
12	TRINITY_DN3	0.27	2e−16	1e−15	20 x 17	
13	TRINITY_DN5	−0.38	2e−16	1e−15	1 x 1	
14	TRINITY_DN1	0.42	2e−16	1e−15	14 x 3	
15	TRINITY_DN7	0.26	2e−16	1e−15	15 x 5	
16	TRINITY_DN5	0.28	2e−16	1e−15	14 x 4	
17	TRINITY_DN4	1.16	2e−16	1e−15	15 x 3	
18	TRINITY_DN4	−1.07	2e−16	1e−15	1 x 1	
19	TRINITY_DN4	−0.46	2e−16	1e−15	18 x 20	
20	TRINITY_DN4	0.3	2e−16	1e−15	12 x 5	

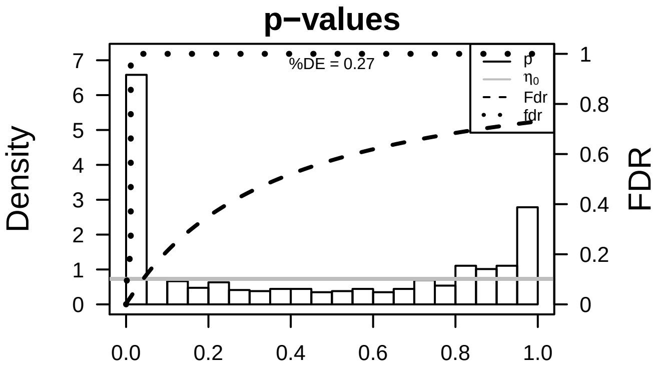

Supplement: Supplementary file 1 [file cells-09-00779-s001.zip › Supplementary materials/FileS3.zip!/FileS2/Hormones - Results/Summary Sheets - Samples/Zhongwan.6.10.4..pdf]
